# Supplementary material for: Synergistic Combination of Irinotecan and Rapamycin Orally Delivered by Nanoemulsion for Enhancing Therapeutic Efficacy of Pancreatic Cancer
Source: Pharmaceutics. 2023 Jan 31;15(2):473. doi: 10.3390/pharmaceutics15020473 (PMC9963937; doi:10.3390/pharmaceutics15020473)
Supplement: Supplementary file 1 [file pharmaceutics-15-00473-s001.zip › pharmaceutics-2039551-supplementary.pdf]

*pharmaceutics*

MDPI

# Synergistic Combination of Irinotecan and Rapamycin Orally Delivered by Nanoemulsion for Enhancing Therapeutic Efficacy of Pancreatic Cancer

**Yu-Hsuan Liu, Ling-Chun Chen, Wen-Ting Cheng, Pu-Sheng Wei, Chien-Ming Hsieh, Ming-Thau Sheu, Shyr-Yi Lin, Hsiu-O Ho and Hong-Liang Lin**

## Analytical graphs

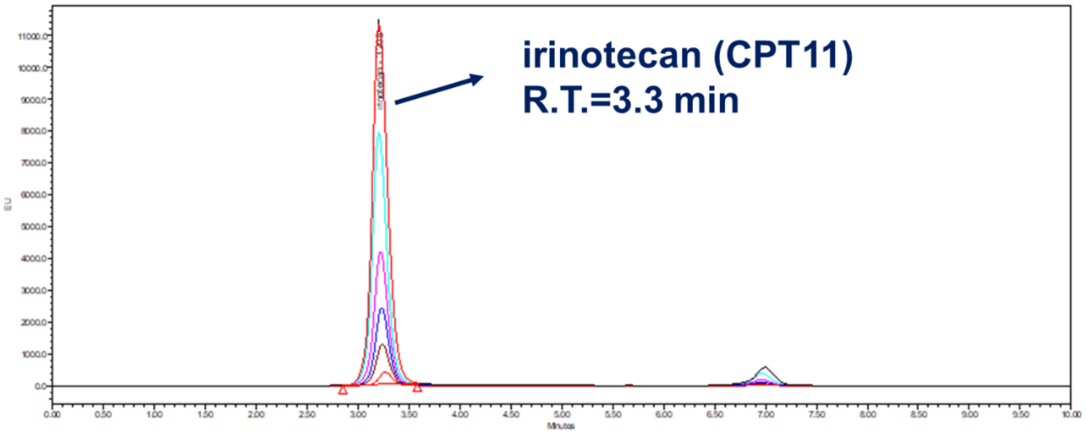


**(A)**

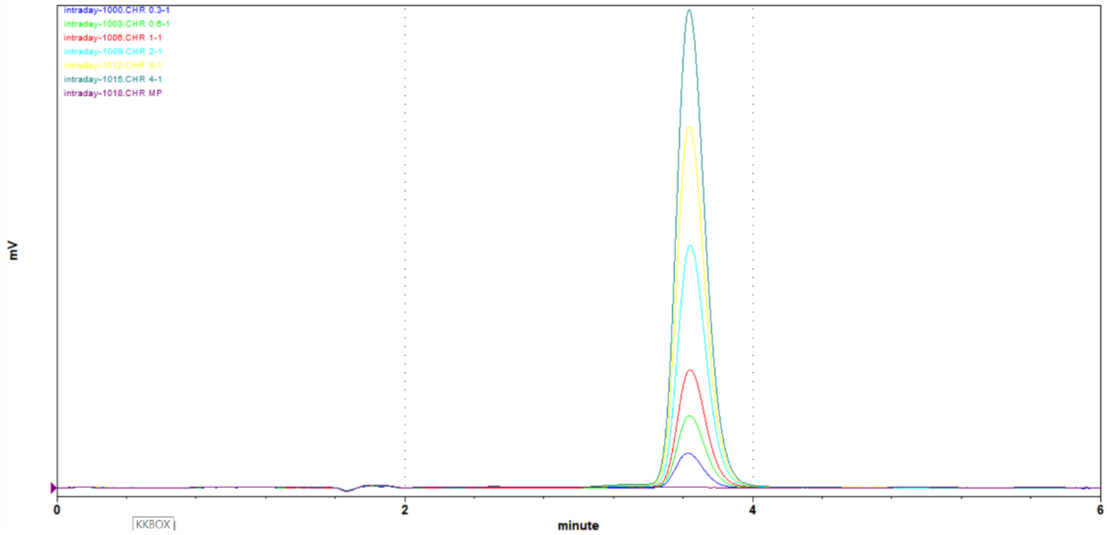


**(B)**

**Figure S1.** Analytical graphs of HPLC: (**A**) irinotecan, and (**B**) rapamycin.

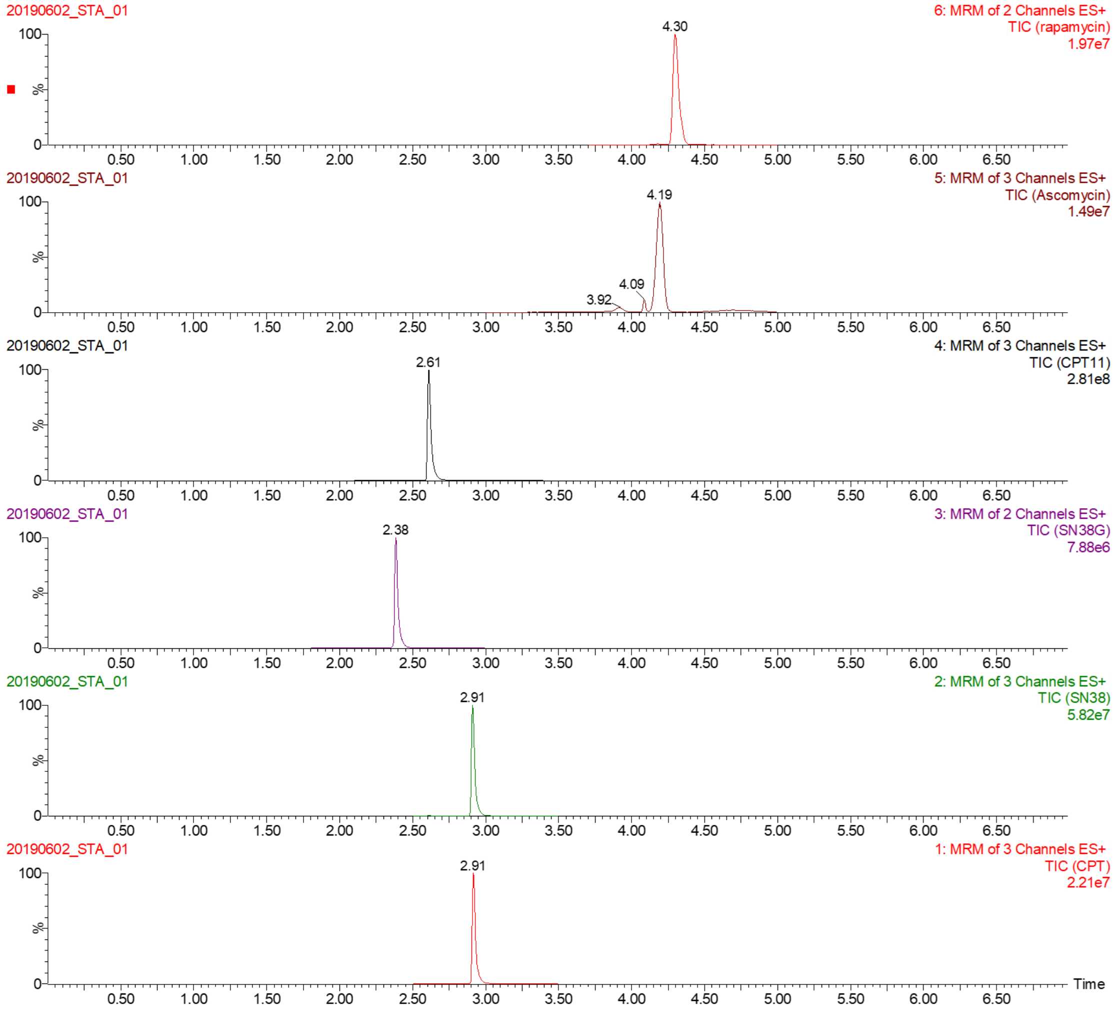


**Figure S2.** Analytical graphs of LCMS.
